# Adapting opioid therapy: real-world analysis of switching from methadone to slow-release morphine and back amid COVID-19 supply chain disruptions

**DOI:** 10.1186/s13722-025-00622-6

**Published:** 2026-01-22

**Authors:** Artūras Barkus, Emilis Subata, Haris Jakavičius, Lina Barkienė, Eglė Lydekaitė, Linas Zdanavičius, Giedrius Likatavičius, Aušra Širvinskienė, Vaida Baltrūnienė

**Affiliations:** 1 Department of Pathology and Forensic Medicine, Institute of Biomedical Sciences, Faculty of Medicine, Vilnius University, M. K. Ciurlionio str. 21, Vilnius, 03101 Lithuania; 2 Clinic of Psychiatry, Institute of Clinical Medicine, Faculty of Medicine, Vilnius University, Vilnius, 03101 Lithuania; 3 Department of Sociology, Institute of Sociology and Social Work, Faculty of Philosophy, Vilnius University, Vilnius, Lithuania

**Keywords:** Methadone, COVID-19, Opioid use disorder, Slow-release oral morphine, Addiction treatment, Clinical outcomes

## Abstract

**Background:**

This study evaluated the effectiveness and patient outcomes of a temporary switch from methadone to slow-release oral morphine (SROM) during COVID-19-related supply disruptions in Lithuania in 2022.

**Methods:**

Data from 231 patients at the Vilnius Branch of the Republican Centre for Addictive Disorders who received SROM for at least two days were retrospectively analyzed. The key metrics included methadone and SROM dosages, withdrawal severity (Clinical Opioid Withdrawal Scale (COWS)), and retention rates at 1, 3, 6, and 12 months post-switch. The data were compared by sex, methadone dosage group (low: 10–60 mg/d, medium: 61–100 mg/d, high: 101–150 mg/d), and clinic attendance frequency. To contextualize long-term outcomes, retention rates were compared with annual program-level data from 2018 to 2024.

**Results:**

Patients received SROM for an average of 8.4 days at an initial methadone-to-SROM ratio of 1:4, which increased to 1:5.23. Withdrawal symptoms were generally mild, peaking at a mean COWS score of 8.2. Women experienced more severe symptoms than men did. After two weeks of SROM therapy, methadone supplies were restored, and patients resumed their original treatment. The retention rates remained high at 1, 3, 6, and 12 months (97.8%, 96.1%, 93.5%, and 89.2%, respectively), with higher retention rates among patients with take-home doses, higher baseline methadone dosages, or longer treatment histories. Long-term program data confirmed that the temporary switch to SROM did not adversely affect overall treatment engagement compared with preceding and subsequent years.

**Conclusions:**

A temporary switch to SROM effectively managed methadone supply disruptions by serving as a viable substitute for methadone, causing minimal withdrawal symptoms and maintaining long-term retention. Coordinated clinical monitoring, institutional protocols, and supportive policy measures ensure continuity of care, emphasizing the value of flexible, personalized treatment strategies during crises.

## Introduction

The COVID-19 pandemic disrupted healthcare systems worldwide and placed particular strain on opioid agonist treatment (OAT), leading to reduced program availability in many settings [1, 2] and, in some cases, disruptions in the supply of opioid use disorder (OUD) medications [3].

This global pattern was mirrored in Lithuania, which faced a nationwide methadone shortage in January 2022, when the Republican Centre for Addictive Disorders (RCAD) – the country’s central supplier of methadone – encountered supply chain disruptions. As the RCAD procures methadone centrally and distributes it to its branches across five cities and to primary healthcare centers for addiction treatment, this shortage affected more than 400 patients nationwide. At the time, methadone was the primary medication prescribed for long-term OUD treatment in Lithuania. The RCAD explored the possibility of temporarily importing methadone from neighboring Latvia; however, despite good professional contacts, this proved unfeasible, as the same regional supplier served the Baltic states, which were also facing potential supply disruptions. Because import licenses can only be issued through private distributors rather than public institutions, additional methadone supplies could not be secured in time.

This critical situation necessitated rapid, coordinated solutions to prevent treatment interruption and adverse outcomes. In response, the RCAD temporarily offered alternative medications, including buprenorphine/naloxone and slow-release oral morphine (SROM).

SROM has gained recognition as a viable OAT option in several European countries [4–6]. While methadone has traditionally been the preferred OAT medication because of its long half-life, SROM has a shorter half-life and is sometimes split-dosed in practice; however, most clinical guidelines and studies support its use once daily in OAT programs [7–16]. Although early systematic reviews [17, 18] offered limited evidence of its effectiveness compared with methadone, more recent research has provided stronger support. A meta-analysis confirmed that SROM is comparable to methadone in terms of patient retention and reduction in heroin use, with potential benefits in reducing cravings and similar safety outcomes [19]. Both randomized controlled trials and long-term observational studies suggest that SROM is as effective as methadone while potentially offering additional advantages, such as a better safety profile, reduced cravings, and improvements in mental and physical health [4, 7–9, 20]. Nonetheless, most studies have been small-scale, relatively short in duration, and often involve patients who have not responded well to methadone.

Given these emerging findings and the urgent need for a substitute medication, Lithuanian authorities swiftly addressed the impending methadone shortage. In mid-December 2021, the supplier company issued an official warning regarding a forthcoming 3–4 week supply disruption due to delays in obtaining export licenses. In response, the Ministry of Health amended the legal framework within only two weeks, allowing the use of SROM as a temporary substitute for methadone despite SROM’s registered indication being solely for pain management rather than OUD treatment. As the national reserves of SROM were also limited, parallel efforts were made to estimate whether the available stock would suffice or if suppliers would need to obtain emergency import licenses from neighboring countries.

This unique scenario offered an opportunity to investigate whether an abrupt, short-term switch from methadone to SROM, followed by a return to methadone, could affect patient outcomes. Specifically, we examined dosing and conversion dynamics, withdrawal trajectories, time to stabilization, and methadone retention up to 12 months after the program-wide transition.

## Methods

### Study objectives

The primary objective of this study was to evaluate whether the short-term switch from methadone to SROM, followed by a return to methadone, affected patient retention rates at 1, 3, 6, and 12 months.

The secondary objectives were as follows:

- To identify potential factors associated with more severe withdrawal symptoms and a lower likelihood of remaining in methadone treatment.
- To determine the final stable methadone-to-SROM ratio.

### Participants

This retrospective cohort study analyzed data from individuals who received methadone therapy at the Vilnius Branch of the Republican Centre for Addictive Disorders (RCAD) in Lithuania and switched to SROM for at least two days following COVID-19-related methadone supply disruptions in January 2022. Although all RCAD branches in Lithuania implemented the switch to SROM, we selected data from the Vilnius Branch for analysis because its systematic collection processes likely reduced potential bias and ensured higher data reliability.

### Intervention (Methadone – SROM transfer procedure)

Given the urgency of this situation, the RCAD developed a local clinical protocol to facilitate a smooth medication change, which was promptly reviewed and approved by the internal ethics committee.

Patients were informed about the impending disruption of methadone supply and the need for alternative medications at the time of their routine clinic visits. They were offered two options: SROM or buprenorphine/naloxone (BNX); however, none of the patients chose BNX. Based on international guidelines and previous research suggesting a methadone-to-SROM conversion ratio of 1:4 to 1:8 [7–16, 21], the RCAD’s local protocol recommended an initial 1:4 ratio, mainly owing to limited SROM availability. The SROM formulation used during this period consisted of film-coated, extended-release tablets available in strengths ranging from 10 to 200 mg.

Patients began SROM treatment during their usual clinic visit schedule. Those who still possessed take-home methadone doses at home started the switch only after completing their remaining methadone supply. Consequently, the duration of SROM use varied between individuals, according to the timing of initiation and the restoration of methadone availability. Once methadone became available again, all patients were switched back promptly, typically to their pre-switch dosage, unless individual adjustments were required. Continuing treatment with SROM was not offered as an option.

All patients, including those who had previously received take-home methadone, were initially required to attend daily follow-up visits to monitor withdrawal symptoms and adjust SROM dosages as needed. As the shortage of methadone continued, exemptions were granted on a case-by-case basis when daily attendance created excessive burden, for example due to employment obligations or significant comorbidities. Eligibility for take-home methadone at RCAD is based on institutional criteria requiring a stable social and somatic condition, negative urine toxicology for illicit substances, and consistent adherence to the treatment plan. Additional justifications may also be considered. Each decision is jointly made by the case manager and the treating physician, remains valid for one to three months, and allows up to six consecutive take-home doses, all documented in the patient’s pharmacotherapy card. These criteria, established for methadone before the shortage, were applied in a similar way when limited take-home SROM exemptions were considered.

SROM was prescribed once daily under supervision, consistent with published guidelines and clinical trials that have evaluated SROM in OAT [7–16]. Routine evening or split take-home doses were not permitted due to legal restrictions, diversion concerns, and limited supply. Although the protocol specified once-daily administration, informal reports indicated that some patients divided their take-home doses during weekends. These occurrences were not systematically recorded and did not modify the overall once-daily dispensing policy.

During the switch, each patient was monitored daily, and their health status was documented in medical records. Patients were encouraged to present at their usual time, typically in the morning, to ensure consistency in assessments, although some variability remained. Withdrawal severity was assessed using the Clinical Opioid Withdrawal Scale (COWS), with measurements taken at every clinic visit just before administering the prescribed dose. SROM dosages were adjusted accordingly, with the goal of maintaining a COWS score of 6 or lower. Once withdrawal symptoms stabilized, the SROM dosage remained unchanged until the methadone supply was restored.

A small number of patients were not switched to SROM due to circumstances requiring continued methadone delivery outside the clinic. These included hospitalizations (e.g., tuberculosis or other acute conditions) and situations necessitating home methadone delivery due to chronic obstructive pulmonary disease or confirmed COVID-19 infection. In addition, SROM was not prescribed to pregnant patients, those on very low methadone doses (< 10 mg), or individuals engaged in safety-sensitive occupations involving heavy machinery. One patient explicitly declined SROM initiation and voluntarily discontinued treatment. These individuals were excluded from the analytical sample described in the Results section.

### Data collection

Data were extracted from patients’ clinical records and included age, sex, duration of methadone treatment prior to the switch (calculated from the recorded initiation date of methadone therapy), baseline methadone dose (categorized as low, 10–60 mg/day; medium, 61–100 mg/day; or high, 101–150 mg/day), and take-home methadone status at baseline. SROM-related variables included dosage and adjustments, duration of SROM treatment, and time to stabilization of the SROM dose. Withdrawal severity was assessed using the Clinical Opioid Withdrawal Scale (COWS). Program retention was evaluated at 1, 3, 6, and 12 months (defined as receiving an opioid agonist dose on days 30, 90, 180, and 365 following the last dose of SROM). Methadone dosage consistency following the return to standard treatment was also recorded.

### Statistical analysis

Comparisons were made between groups based on sex, methadone dosage category (low, medium, or high), and clinic attendance frequency (daily visits versus take-home doses). Nominal variables were analyzed using the chi-square test, and continuous variables were examined using the independent-samples t test or Mann–Whitney U test for nonparametric data. Correlation analyses (Pearson’s r and Spearman’s ρ) were performed to assess relationships between treatment duration, withdrawal severity (COWS scores), methadone-to-SROM conversion ratios, and program retention. Linear regression analyses were conducted to examine associations between variables and to assess temporal trends in program-level indicators across years. A two-tailed p value of < 0.05 was considered statistically significant. All statistical analyses were performed using Jamovi software, version 2.3 [22].

### Program-level contextual analysis

To contextualize retention outcomes observed during the methadone-to-SROM switch, aggregate annual data from the RCAD methadone program were reviewed for the years 2018–2024. Institutional end-of-year reports provided information on the total number of patients treated, new admissions, and those remaining in treatment on December 31 of each year. Annual retention was calculated as the proportion of patients active at year-end relative to the total number treated during that year.

## Results

### Baseline patient characteristics

Data from 231 patients at the Vilnius branch of the RCAD who received SROM for at least two days were analyzed. An additional 15 patients remained on methadone during the shortage period and were not switched to SROM due to medical or logistical reasons, as described in the Methods. Table 1 outlines the baseline characteristics of the Vilnius cohort before switching to SROM. The cohort was predominantly male, comprising three-quarters of the sample. Approximately half of the patients attended the clinic daily, whereas the other half received take-home doses of methadone. The average methadone dose before switching to SROM was 77.8 mg (SD ± 26 mg). Patients had been in methadone treatment for a mean of 5.5 years (SD ± 4.8; median 4.2; range 0–23 years) prior to the switch.

**Table 1:** Characteristics of patients before SROM treatment

Characteristic	*N* (%)
**Sex** (***N*** = 231)	
Male	176 (76.2%)
Female	55 (23.8%)
**Clinic attendance** (***N*** = 231)	
Daily	121 (52.4%)
Take-home doses	110 (47.6%)
**Methadone doses before switch** (***N*** = 231)	
Low (10–60 mg/d.)	76 (32.9%)
Medium (61–100 mg/d.)	117 (50.6%)
High (101–150 mg/d.)	38 (16.4%)

### Methadone-to-SROM switch: dosing and conversion dynamics

Patients underwent SROM treatment for an average of 8 days (range: 2–13). Following the treatment protocol, clinicians prescribed the initial SROM dosage at a 1:4 ratio to methadone, yielding a mean initial dose of 312 mg (SD ± 121 mg). By the end of the treatment period, the mean methadone-to-SROM conversion ratio had increased to 1:5.23 (SD ± 1.27). We found no significant differences in the final conversion ratio by sex, take-home dosing status, or initial methadone dose (one-way ANOVA). A weak but significant negative correlation was found between the final conversion ratio and years in methadone treatment (*r* = − 0.16, *p* = 0.017), indicating that patients with longer treatment histories required proportionally lower SROM doses to achieve stabilization (Tables 2 and 3).

**Table 2:** Summary of descriptive statistics for methadone and SROM dosing and conversion dynamics

	*N*	Mean	Median	Standard deviation	Minimum	Maximum
Years in the methadone treatment	215	5.45	4.17	4.79	0 (10 days)	23.2
Initial methadone dose (mg)	231	77.8	80	29.6	10	150
Initial SROM dose	231	312	320	121	40	620
Duration of SROM treatment (days)	231	8.4	9	2.6	2	13
SROM dose on final day (mg)	231	407	400	180	0	900
Final Methadone to SROM ratio	231	1: 5.23	1: 5	1: 1.27	1: 4	1: 14.3
Day of last SROM dose adjustment	231	4.2	3	2.6	1	9

On average, patients reached the effective SROM dose after 4.2 days (SD ± 2.6), meaning that no further adjustments were made after this point. We found no significant sex-based differences in the time to reach the effective SROM dose.

However, those who did not previously receive take-home methadone needed more time to stabilize their SROM dose than those who did (mean [SD]: 4.5 [± 2.7] vs. 3.8 [± 2.4] days, *p* = 0.020). Time in methadone treatment correlated weakly and negatively with the day of last SROM adjustment (*r* = − 0.15, *p* = 0.030), indicating that patients with longer treatment histories stabilized sooner.

One-way ANOVA revealed that patients on low methadone doses stabilized faster than those on medium or high doses did (mean [SD]: 3.3 [± 2.4] vs. 4.6 [± 2.6] and 4.5 [± 2.6] days, respectively; *p* = 0.002).

### Assessment of withdrawal severity during SROM treatment

On the first day after methadone cessation, before starting SROM, patients reported minimal or no withdrawal symptoms, with a mean COWS score of 2.4 (SD ± 2.98). As per the treatment protocol, the SROM dose was increased by 20% if the COWS score exceeded 6. On day 2, patients experienced more pronounced withdrawal symptoms, with an average COWS score of 6.3 (SD ± 4.37). The most severe withdrawal symptoms occurred, on average, 3.2 days (SD ± 1.95) after starting SROM, with the highest recorded COWS scores averaging 8.2 (SD ± 4.41).

On day 2, women reported an average COWS score of 7.37, which was significantly higher than the average score for men, which was 5.96 (*p* = 0.039). Additionally, women experienced higher maximum COWS scores, averaging 9.49, compared to men’s average of 7.78 (*p* = 0.012).

One-way ANOVA revealed significant differences in withdrawal symptoms on day 2 among the low, medium, and high methadone dose groups, with mean (SD) COWS scores of 5.3 (± 4.2), 7.1 (± 4.3), and 6.2 (± 4.5), respectively (*p* = 0.02). Additionally, patients on lower methadone doses reported significantly lower maximum COWS scores than did those on medium and high doses (mean [SD]: 6.6 [± 4.3] vs. 9.0 [± 4.3] and 8.7 [± 4.3], respectively; *p* < 0.01). A weak negative correlation was observed between years in methadone treatment and maximum COWS score (*r* = − 0.15, *p* = 0.025), indicating that patients with longer treatment histories experienced slightly milder withdrawal symptoms. No significant correlation was found between time in treatment and COWS scores on day 2 (*r* = − 0.12, *p* = 0.085).

We found no significant differences in withdrawal severity for other variables.

**Table 3:** Withdrawal outcomes during SROM treatment

	*N*	Mean	Median	Standard deviation	Minimum	Maximum
COWS score on day 1	231	2.41	1	2.98	0	16
COWS score on day 2	220	6.30	7	4.37	0	18
Day of peak COWS score	222	3.20	2	1.95	1	9
Maximum recorded COWS score	231	8.19	8	4.41	0	24

### Return to methadone and retention rates after SROM therapy

After 8.4 days (on average) of SROM therapy, all patients returned to methadone treatment following standard practice. Most (87%) resumed their pre-transfer methadone dosage within one week, although some required individual adjustments. One patient, who previously used a low methadone dose (25 mg), chose to discontinue opioid agonist treatment entirely during the SROM phase.

**Fig. 1:**
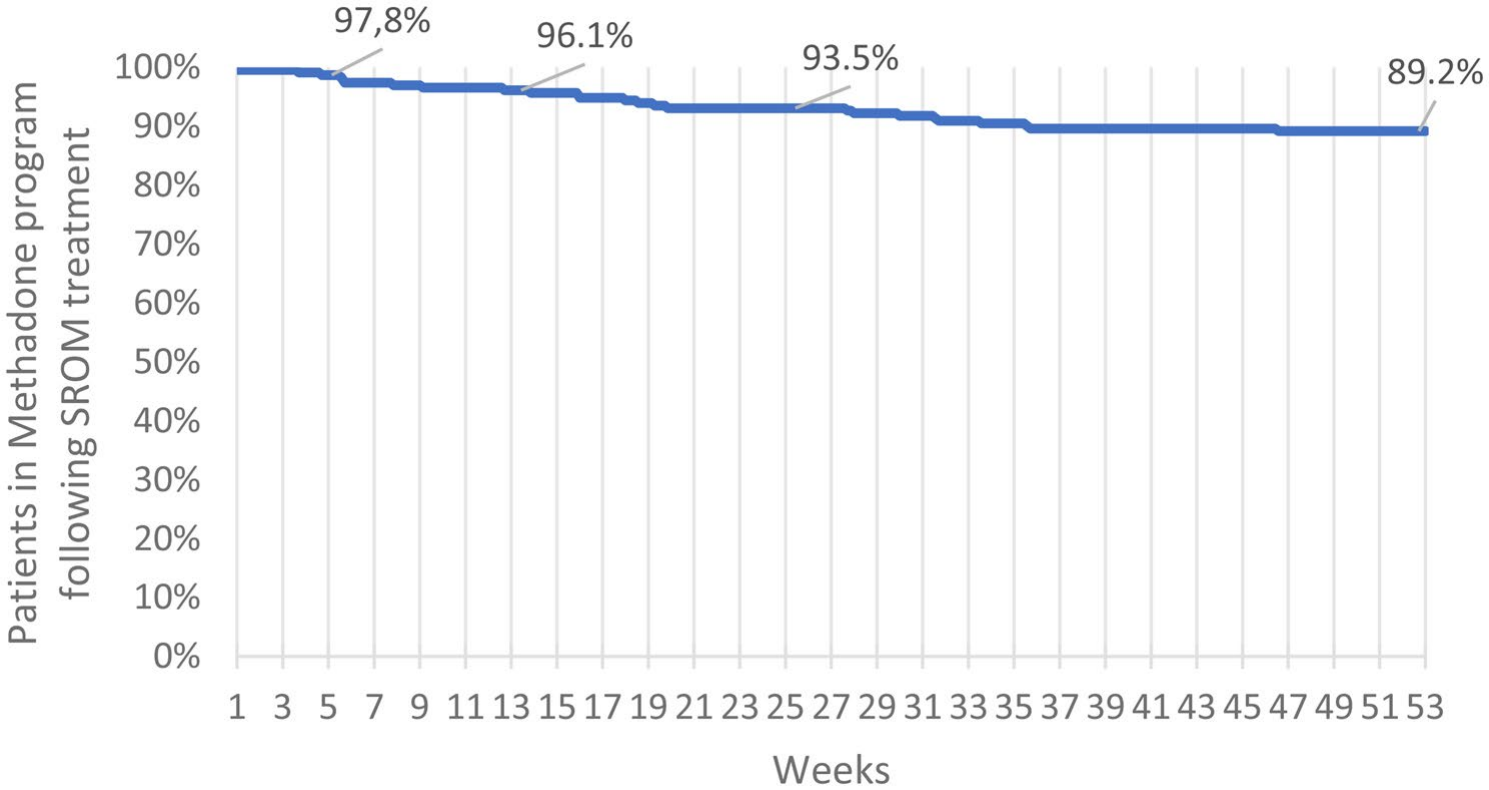
Patient retention rates in the methadone program following SROM treatment

Overall retention in methadone therapy remained high following SROM, with rates of 97.8% at 1 month, 96.1% at 3 months, 93.5% at 6 months, and 89.2% at 12 months (Fig. 1; Table 4). We observed no significant sex-based differences in retention. However, patients eligible for take-home methadone had greater long-term retention than those who attended the clinic daily (98.2% vs. 89.3% after 6 months, *p* = 0.006; 97.3% vs. 81.8% after 12 months, *p* < 0.001). We also found a week but significant positive correlation between time in methadone treatment and program retention duration after SROM treatment (*r* = 0.24, *p* < 0.001), indicating that patients with longer treatment histories were more likely to remain engaged in care.

**Table 4:** Overview of retention rates and methadone dosage changes following SROM treatment

Characteristic	*N* (%)
**Retention rates in the Methadone program:**	
After 1 month	226 (97.8%)
After 3 months	222 (96.1%)
After 6 months	216 (93.5%)
After 12 months	206 (89.2%)
**Methadone Dosage Consistency (Dose Unchanged from Pre-SROM Treatment)**	
Upon Return to Methadone	211 (91.3%)
One Week Post-Return	201 (87.4%)

An initially lower methadone dose was associated with lower retention at 6 months (mean of 62 mg [SD ± 25.1] vs. 78.9 mg [SD ± 29.6], *p* = 0.032). At 12 months, retention rates were 87% (SD ± 34%), 88% (SD ± 33%), and 97.4% (SD ± 16%) among those receiving low, medium, and high methadone doses, respectively (*p* = 0.025).

Patients with a lower methadone-to-SROM ratio consistently demonstrated better retention across multiple time points (Table 5).

Program-level data from the Vilnius branch of the RCAD methadone program for 2018–2024 are summarized in Table 6. During this period, the total number of patients treated annually ranged from 351 to 395, with 80–130 new admissions each year. Year-end retention, calculated as the proportion of patients active in treatment on December 31 relative to all treated during that year, ranged from 69% to 77%. Statistical analyses showed no significant differences in retention proportions between years (χ²[6] = 10.97, *p* = 0.089) and no significant Temporal trend (β = − 0.0065 per year, *p* = 0.31)

**Table 5:** Retention rates in the methadone program following SROM treatment and methadone-to-SROM conversion Ratios

Methadone-to-SROM Ratio Independent samples t test	Discontinued Treatment	Continued Treatment	*p* values
3 Months Retention	1:6.5 (SD ± 2.4)	1:5.2 (SD ± 1.2)	0.002
6 Months Retention	1:5.9 (SD ± 2.0)	1:5.2 (SD ± 1.2)	0.025
12 Months Retention	1:5.7 (SD ± 1.8)	1:5.2 (SD ± 1.2)	0.04

**Table 6:** Annual Program-Level data from the Vilnius branch of the RCAD methadone maintenance Program, 2018–2024

Year	Total treated	New admissions	Active at year-end	Retention*
2018	358	92	266	74%
2019	351	85	246	70%
2020	361	87	279	77%
2021	362	83	274	76%
2022	356	82	263	74%
2023	377	114	267	71%
2024	395	128	272	69%

*Retention calculated as active patients on December 31 / total treated during the year

## Discussion

In this program-wide, short-term switch from methadone to slow-release oral morphine (SROM) and back, continuity of care remained high and clinical stabilization was rapid under daily monitoring. The mean duration of SROM use was 8 days (range 2–13). This variability reflected operational realities rather than clinical instability, as some patients still had take-home methadone and therefore started SROM later, and all were switched back immediately once methadone became available. Day-2 withdrawal was modest (mean COWS 6.3), peak withdrawal was low–moderate (mean maximum COWS 8.2), and most patients stabilized by day 4. Twelve months after resuming methadone, 89.2% of patients remained in treatment. A few individuals experienced more pronounced withdrawal symptoms (maximum COWS scores up to 24) or reported practical difficulties related to daily attendance, but these cases were isolated and transient, without measurable effects on retention or overall stability. Although the shortage of methadone itself lasted only about two weeks, we followed outcomes to 12 months because even brief OAT interruptions can destabilize care [23]. Direct comparisons with prior studies are limited because most evaluate newly initiating patients, who typically show lower retention [24–26]; nonetheless, in this established cohort, a brief SROM switch did not appear to disrupt ongoing engagement.

Our final conversion ratio (≈ 1:5.2) aligns with prior reports clustering around ~ 1:5 [10, 12, 27], though some studies describe higher requirements (1:6–1:8 or higher) [7–9, 14, 15]. The upward adjustment from 1:4 toward ~ 1:5 over several days mirrors clinical guidance to titrate during induction [10, 11, 21, 28, 29]. Stabilization occurred faster here (≈ 4 days) than in several prior reports (6–14 days) [9, 10, 12, 16]. Patients on lower baseline methadone doses stabilized more quickly and experienced milder withdrawal, consistent with less complex opioid requirements. Conversely, higher final conversion ratios were modestly associated with lower long-term retention, possibly reflecting more severe dependence or psychosocial instability. Methadone’s pharmacokinetics can complicate direct dose conversions because of substantial interindividual variability in CYP-mediated metabolism (particularly inducible CYP2B6), genetic polymorphisms, drug–drug interactions, and incomplete cross-tolerance [30–33]. Nevertheless, our data did not support nonlinear conversion dynamics: the final methadone-to-SROM ratios were comparable across baseline methadone dose categories, likely reflecting the standardized dosing protocol, close clinical monitoring, and the brief duration of the switch period.

Withdrawal symptoms were generally mild and transient, with an average peak COWS score of 8.2 that resolved within several days under daily supervision. Women exhibited slightly higher COWS scores than men, but as subjective and objective components were not differentiated, further interpretation is limited. Longer prior participation in methadone treatment correlated with faster stabilization, milder withdrawal, and higher long-term retention, reflecting the greater stability of established patients. Similarly, eligibility for take-home methadone, which indicates clinical and social stability, was associated with better retention after the switch. This likely reflects selection rather than a causal effect of take-home dosing, as such patients tend to be more adherent and engaged in treatment.

SROM was dispensed once daily under observation, consistent with OAT guidelines and trials. Routine split dosing was not implemented because of legal restrictions, diversion concerns, and limited supply. A few patients informally reported dividing weekend take-home doses, but this was not systematic and did not alter the once-daily policy. Patients were informed about the switch during their regular clinic visits rather than in advance. While advance communication might be preferable under routine circumstances to allow more preparation, in this crisis setting immediate on-site notification was considered the safer approach to prevent potential destabilization before the switch.

No overdoses or serious adverse events were recorded; one unrelated death occurred due to pre-existing illness. Three formal complaints cited the burden of daily attendance, highlighting logistical rather than clinical challenges. However, we could not retrieve systematic data on adverse events, diversion, or attendance patterns, so unreported incidents cannot be excluded.

The effective management of this temporary switch reflected coordination at multiple levels: rapid regulatory action by the Ministry of Health, local protocol development by the RCAD, and daily clinical oversight to adjust dosing and monitor withdrawal. This experience highlights the importance of regulatory flexibility, predefined emergency procedures, and strong communication between policymakers and clinicians during treatment crises.

This single-site, retrospective analysis has several limitations. The study design limited systematic capture of safety and adherence data, and associations (e.g., with take-home status or treatment duration) are observational. We lacked detailed information on concurrent substance use, psychiatric comorbidities, and other confounders that may have influenced withdrawal or retention outcomes, and we could not exclude unreported adverse events among patients who discontinued or were lost to follow-up. Data on SROM misuse or diversion were not systematically collected. Limited SROM availability during the shortage may also have constrained dosing flexibility. The SROM formulation (film-coated, extended-release tablets) and national legal context may differ from other settings, and because this was a rapid, crisis-driven switch rather than a planned transition, generalizability is restricted. Future prospective, multicenter studies should examine safety, conversion dynamics, and patient experience during both planned and emergency medication substitutions.

## Conclusion

The temporary substitution of methadone with SROM during COVID-19-related supply disruptions effectively maintained OAT continuity, achieving high retention rates and minimal clinical disruption. Coordinated policy, institutional, and clinical responses were crucial to the success of this intervention, underscoring the importance of multilevel planning and rapid decision-making. This study highlights the potential of SROM as a viable alternative during methadone supply crises and emphasizes the need for healthcare providers to develop individualized treatment plans to optimize long-term outcomes.

## Data Availability

No datasets were generated or analysed during the current study.
